# Role of estrogen receptor coregulators in endocrine resistant breast cancer

**DOI:** 10.37349/etat.2021.00052

**Published:** 2021-08-30

**Authors:** Kristin A. Altwegg, Ratna K. Vadlamudi

**Affiliations:** 1 Department of Obstetrics and Gynecology, University of Texas Health San Antonio, San Antonio, TX 78229, USA; 2 Mays Cancer Center, University of Texas Health San Antonio, San Antonio, TX 78229, USA; University of Edinburgh, UK

**Keywords:** Estrogen receptor, coregulators, transcriptional activation, estrogen, hormonal action, signal transduction, endocrine therapy resistance

## Abstract

Breast cancer (BC) is the most ubiquitous cancer in women. Approximately 70–80% of BC diagnoses are positive for estrogen receptor (ER) alpha (ERα). The steroid hormone estrogen [17β-estradiol (E2)] plays a vital role both in the initiation and progression of BC. The E2-ERα mediated actions involve genomic signaling and non-genomic signaling. The specificity and magnitude of ERα signaling are mediated by interactions between ERα and several coregulator proteins called coactivators or corepressors. Alterations in the levels of coregulators are common during BC progression and they enhance ligand-dependent and ligand-independent ERα signaling which drives BC growth, progression, and endocrine therapy resistance. Many ERα coregulator proteins function as scaffolding proteins and some have intrinsic or associated enzymatic activities, thus the targeting of coregulators for blocking BC progression is a challenging task. Emerging data from *in vitro* and *in vivo* studies suggest that targeting coregulators to inhibit BC progression to therapy resistance is feasible. This review explores the current state of ERα coregulator signaling and the utility of targeting the ERα coregulator axis in treating advanced BC.

## Introduction

Globally, breast cancer (BC) is the leading cause of cancer-related mortality in females and thus accounts for approximately 684,996 deaths annually [1]. BC is a complex and highly heterogeneous disease and is composed of distinct subtypes associated with different clinical outcomes [2]. These subtypes are based on the expression of estrogen receptor (ER) alpha (ERα), the progesterone receptor (PR), and the human epidermal growth factor receptor-2 (HER2)/neu. Molecular analysis through gene expression profiling of tumors revealed four intrinsic BC subtypes: luminal ERα positive (ERα+; luminal A and luminal B), HER2 enriched, and basal-like [triple-negative BC (TNBC)] [3, 4]. TNBC lacks ERα, PR, and HER2 [5].

The steroid hormone, estrogen [17β-estradiol (E2)], plays an integral role in the development of normal breast tissue. Further, E2 can also function as a driver in the initiation and progression of BC. The majority of BC starts as hormone-dependent; approximately 70–80% of BC diagnoses are ERα+, and 55–65% are PR positive (PR+) at the time of initial diagnosis [3]. Patients with HER2 overexpressing BC comprise approximately 15% of all BC diagnoses. TNBC accounts for approximately 15% of all BC and has a poorer prognosis [5–7]. In hormone receptor-positive BC, E2-ERα axis-mediated actions can involve classical genomic signaling, non-classical genomic signaling, and non-genomic signaling. Importantly, the specificity and magnitude of ERα signaling are mediated by interactions between ER and critical coregulators containing a nuclear receptor (NR) box motif [8]. This article will attempt to summarize emerging evidence on the role of coregulators in conjunction with ERα signaling in BC.

## ER and signaling mechanisms

E2 mediates its biological effects using two distinct ERs, ERα and ER beta (ERβ). The estrogen receptor (*ESR*)*1* gene located on chromosome 6 at q25.1 encodes the 66.2 kDa ERα protein. While the *ESR2* gene on chromosome 14 at q23.2 encodes the 59.2 kDa ERβ protein [9]. The first and most well-characterized receptor, ERα was discovered in 1958 by Elwood Jensen, who later established that the estrogen-bound ER migrates to the nuclear compartment to stimulate transcription [10, 11]. The ERα oncogene is the major driver of ~75% of BC, therefore, ERα and ERα-regulated genes serve as therapeutic targets for ERα+ BC. ERβ functions as an anti-proliferative, in many ways antagonizing the function of ERα [12]. ERβ may function as a prognostic marker for tamoxifen resistance [13] and some ERβ isoforms may have oncogenic functions in BC [14]. This review primarily focused and was limited to ERα oncogenic signaling mechanisms in BC.

**Figure 1.**
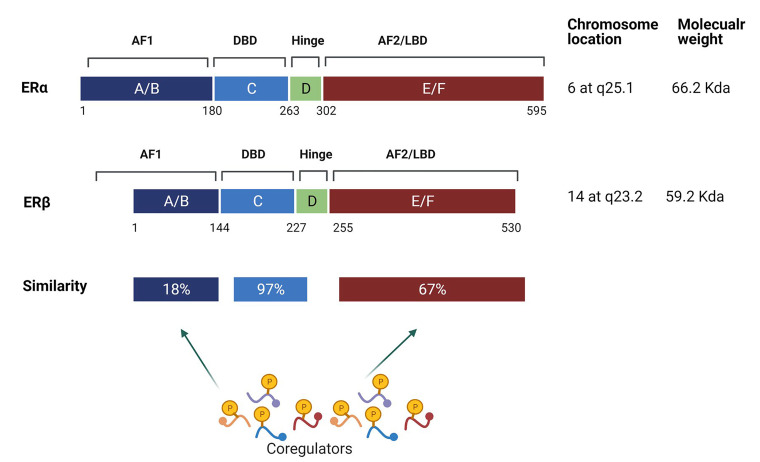
Schematic representation of various domains in ERα and ERβ. ERs consist of six domains labelled A through F. The activation of AF1 region is located in A/B-domain and the DNA binding region is located in the C-domain. The D-domain contains a flexible hinge region. The E-domain contains the ligand-dependent activation of function domain (AF2). The F domain contributes to differences in the activity of ERα and ERβ subtypes and is also involved in receptor interactions with coregulators. Chromosomal localization and similarity between various domains of ERα and ERβ are depicted. The AF1 and AF2 domains facilitate ligand-independent and ligand-dependent interactions with coregulators, respectively. DBD: DNA binding domain

The ERs belong to the family of steroid receptors and contain six functional domains labeled A-F (Figure 1). The *N*-terminal domain (A/B domains, encoded by exon 1) contains the activation of function (AF)1 region which is integral in transcriptional activity. The ERα can be phosphorylated by various kinases, and these phosphorylation events have been established as modulators of ERα activity [15]. The DNA binding domain (C domain, encoded by exons 2–4) contains two zinc-finger motifs. The hinge region (D domain, encoded by exon 4) contains regions for receptor dimerization and nuclear localization. The ligand-binding domain (LBD; E domain) contains the AF2 [16]. This domain participates in several activities including hormone binding, homodimerization and/or heterodimerization, formation of heat-shock protein (HSP) complexes, and transcriptional activation and repression. The *C*-terminal domain (F domain) is encoded by exons 5–8 [16].

Even though ERα and ERβ are structurally similar in modular nature, their LBDs differ in similarity. The DNA binding domains of ERs share 97% similarity, while the AF1 and AF2/LBD domains only share 18% and 67% similarity, respectively (Figure 1). The differences in the amino acid composition of the two ERs are suspected to facilitate the interactions of distinct coregulator proteins by ERs. ERα is the major ER subtype expressed in the mammary epithelium [17]. Further, ERα is implicated as the master regulator and driver of BC progression [8, 18]. ERβ, which was discovered in 1996, has very different if not opposite functions from ERα; thus, it is generally considered a tumor suppressor [19]. Two recent reviews by Zhou et al. [20] and Sellitto et al. [21] covered the role of ERβ in BC and TNBC, respectively. For this review, we only focused on ERα coregulator-driven signaling in ERα hormone action and BC progression.

Published evidence implicates ERα signaling via these four mechanisms. (1) Classical-direct-ER signaling. The classical mechanism of ERα action involves E2 binding to ER, which induces conformational changes in the ERα protein structure. The ligand-bound ERα then forms a dimer and translocates to the nucleus where it binds to specific target genes containing palindromic estrogen response elements (EREs) [22]. (2) Non-classical-indirect-ER signaling. The non-classical mechanism of action involves ERα interactions with other transcription factors (TFs) within the nucleus such as activator protein 1 (Ap1), specificity protein 1 (SP1), etc. and together they activate a different specific set of target genes containing non-ERE or half site-ERE containing genes [23]. Interestingly, approximately one-third of human genes which are regulated by ERα do not contain ERE-like sequences [24], which gives rise to the third mechanism of signaling. (3) Non-genomic-ER signaling. The non-genomic effects of E2-ERα occur rapidly through signaling cascades when ERα interacts with cytosolic kinases such as Src kinase, serine/theronine kinase (Akt), phosphatidylinositol 3-kinase (PI3K), etc. [25–27] and is activated through phosphorylation which gives to distinct genomic outcomes. (4) Lastly, the final mechanism of ERα signaling: ligand-independent ER signaling. ER phosphorylation by oncogenic kinases, or post-translational modifications or mutations of ERα in the LDB contribute to structural changes in ER facilitating activation of ER target genes independent of E2 ligand [28–30]. This mode of signaling is predominant in pathological situations such as endocrine resistant BC.

**Figure 2.**
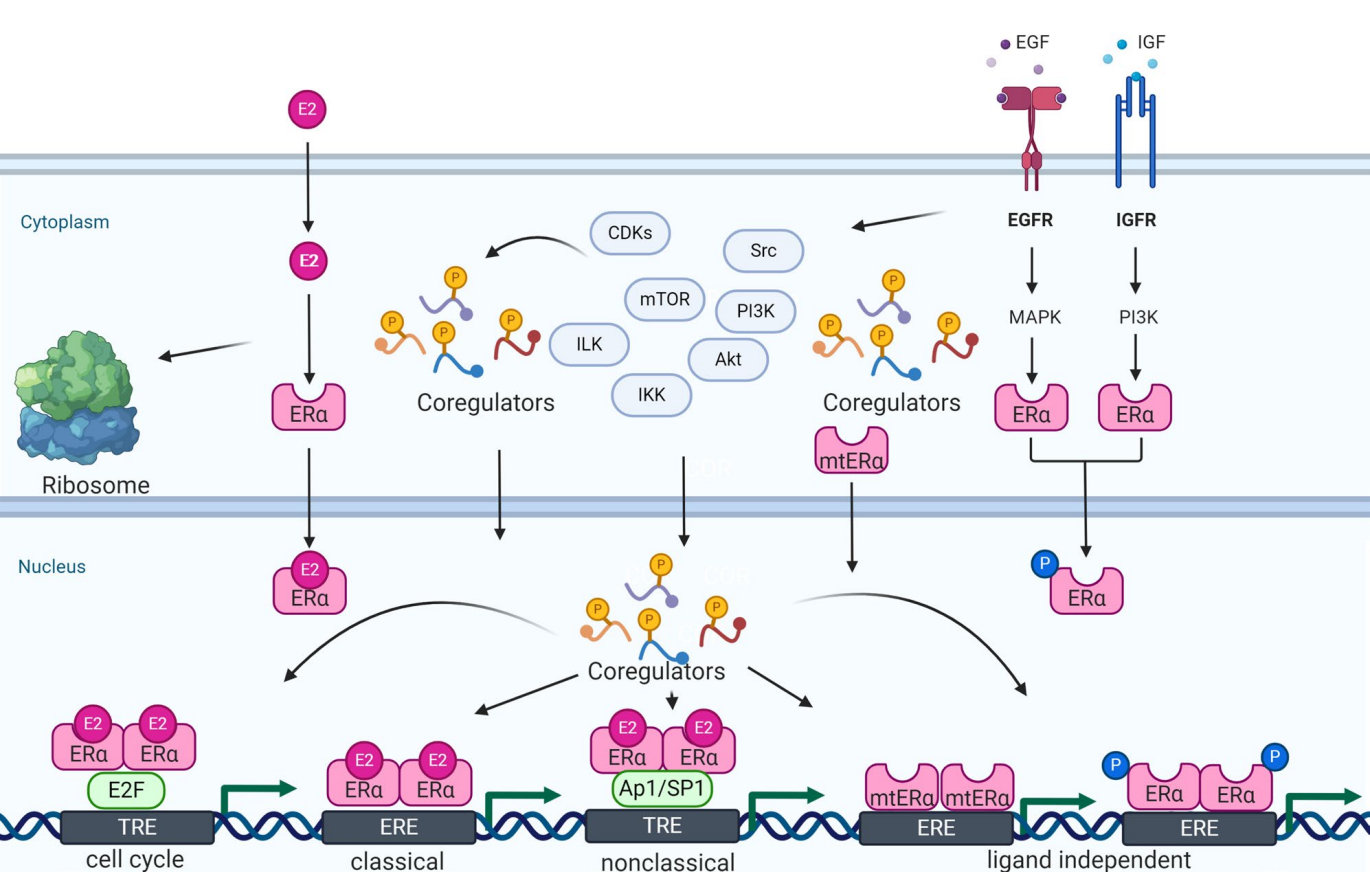
ER coregulator signaling mechanisms. ERα coregulators participate in multiple aspects of ER signaling including ER genomic (classical and non-classical), non-genomic, cell cycle, and ligand-independent signaling. In the cytoplasm, coregulators play a critical role in the activation of ER non-genomic signaling. Coregulators can be phosphorylated by cytosolic kinases and thus function as sensors of non-genomic signaling and integrate that signaling with ER genomic functions. Cell cyclin-dependent kinases (CDKs) also phosphorylate ER coregulators, and thus connect ER signaling to cell cycle progression. ER coregulators play an essential role in both classical and non-classical signaling via liganded ERα by promoting conducive chromatin remodeling. Deregulated growth factor signaling which commonly occurs in BC progression promotes post-translational modifications of ER and its coregulators; this facilitates ligand-independent activation of ERα target genes. Mutant ER (mtER) commonly occur in endocrine therapy resistant BC and mutations in the ligand binding domain of ERα create a constitutively active binding site for coregulators which promotes ligand-independent ER signaling. ILK: integrin-linked kinase; IKK: IkappaB kinase; mTOR: mechanistic target of rapamycin; EGF: epidermal growth factor; EGFR: EGF receptor; MAPK: mitogen-activated protein kinases; IGF: insulin-like growth factor; IGFR: IGF receptor; E2F: early 2 factor; TRE: trehalose; mtERα: mtER alpha

Emerging evidence suggests that the transcriptional activity of ERs is regulated by a diverse array of coregulator proteins called coactivators and/or corepressors [8, 31] (Figure 2). Coactivators preferentially associate with E2 bound ERα, while corepressors associate with antagonist occupied ERα [32, 33]. E2 binding to ERα promotes the formation of multiprotein complexes at ERα target genes to activate transcription [31]. It is widely established that the diverse functions of E2 signaling depend on the differential recruitment of coregulators to the E2 bound ERα [32]. Although the molecular basis of ERα interactions with coregulators is well documented, very little is known regarding the mechanisms by which they influence the development and progression of BC. This review is focused on coregulator signaling in ERα driven BC.

## Coregulator functions in ER genomic actions

Coregulators lack direct sequence-specific recruitment capabilities and their coregulator activity is dictated by NRs, such as ERα, which attract them to the target gene loci. Recruitment of coregulator complexes to the nucleosome is a key step in transcriptional regulation [34]. Coactivator and corepressor complexes are recruited by ER through conserved nuclear receptor box (LxxLL, L is leucine, x is any amino acid) [35] and co-repressor nuclear receptor box (LxxH/IIxxxI/L, L is leucine, H is histidine, I is isoleucine, X is any amino acid) motifs [36], respectively. Coregulators facilitate ERα-mediated transcription by providing diverse enzymatic activities required by the ER for appropriate chromatin modification to achieve optimal transcription [8, 37] (Figure 2). ERα transcriptional outcomes are regulated by the dynamic interactions of histone acetyltransferases and histone deacetylases (HDACs), which are generally associated with coregulators [33]. Coactivators [steroid receptor coactivator (SRC) 1 and amplified in BC 1 (AIB1)] possess histone acetyltransferase activity, while corepressors [NR corepressor (NCOR) and metastasis-associated protein 1 (MTA1)] are associated with HDACs [38]. The ERα interactions with pioneer factors, coregulators, and post-translational modifiers will determine the E2-ERα transcriptional response [39]. Furthermore, BC progression is marked by coregulator-mediated chromatin remodeling and histone modifications [40]. For example, the coregulator, proline glutamic acid and leucine-rich protein 1 (PELP1) has a histone binding domain [41], recognizes histone modifications, and interacts with several chromatin-modifiers including lysine-specific histone demethylase (KDM) 1A [41], HDAC [42], protein arginine methyltransferases (PRMT) [43], and coactivator-associated arginine methyltransferase 1 (CARM1) [44]. Further, these large multiprotein complexes containing coactivators, ERα, and transcriptional regulators assemble at ERα target genes in response to E2 binding to activate transcription [31]. Coregulators are also shown to play a critical role in estrogen-induced chromatin looping interactions during transcription at ER target genes [45]. Collectively, these findings suggest that coregulators are involved in many steps of ERα genomic actions including chromatin modification, remodeling, and transcription changes (Figure 2). Therefore, alterations in coregulators that commonly occur in BC may provide an advantage in enhancing the expression of ERα target genes during BC progression.

## Coregulator functions in ER non-genomic actions

ER-non-genomic signaling is involved in rapid responses to E2 via activation of cytosolic kinases such as Src, MAPK, and PI3K [32] (Figure 2). ERα participates in non-genomic signaling via the formation of a multiprotein complex collectively called a “signalosome” [46]. Mechanistic studies showed that these complexes have several adaptor proteins (caveolins, striatin, p130Cas, Shc, etc.) and coregulator proteins such as SRC3, MTA1s, and PELP1 [47]. Using novel ligands that uniquely activate non-genomic signaling it was demonstrated that non-genomic pathways have distinct biological outcomes [48]. ERα extranuclear actions are also shown to play a role in cell motility/metastasis. PELP1 contributes to ER extranuclear actions leading to cell invasion by modulating the ER-Src-PELP1-ILK 1 (ILK1) pathway [49]. Cytoplasmic PELP1 signaling is shown to stimulate estrogen-related receptor γ (ERRγ) to promote cell survival [50]. E2-ERα signaling utilizes the PELP1-mediated PI3K/Akt signaling cascades to enhance matrix metalloproteinase-9 (MMP-9) expression in ERα+ BC [51]. PELP1/AIB1-containing complexes in the cytoplasm function to promote advanced cancer phenotypes; including outgrowth of stem-like cells which are associated with E2-independent BC progression [52]. Furthermore, PELP1 has been documented to up-regulate pro-tumorigenic IKKɛ and thus enhance the migration of BC cells [53]. It was also demonstrated that PELP1 plays a critical role in the optimal activation of the mTOR, concomitantly, PELP1 deregulation contributes to excessive activation of mTOR signaling [54]. At the plasma membrane, E2 promotes ERα complex formation with HDAC6 and tubulin which contributes to the aggressiveness of ER-positive (ER+) BC cells [55]. In addition, E2-induces SRC-3 phosphorylation via direct interaction with ERα in the cytoplasm; thus indicating it participates in early ER non-genomic actions [56]. The ERα corepressor, MTA1s, sequesters ERα in the cytoplasm, promoting non-genomic signaling, which has been shown to contribute to the malignant BC phenotype [57]. Overall, these findings suggest that coregulator proteins participate in ERα non-genomic actions by connecting ER with cytosolic kinases, and in some instances also by sequestering ER in the cytoplasm. Deregulation of ERα coregulators can excessively activate non-genomic actions in BC cells which may have implications in endocrine therapy resistance.

## Coregulator functions in E2-ERα mediated cell cycle progression

E2-ERα signaling promotes cell proliferation in a wide variety of tissues including the mammary gland [58, 59]. E2 participates in cell cycle progression by promoting activation of CDKs, the hyper-phosphorylation of retinoblastoma (pRb) in ERα+ BC cells [59], and utilizing coregulator proteins such as PELP1 to couple E2-ER signaling to the cell cycle machinery. PELP1 is a unique substrate of CDKs and is necessary for E2 mediated progression through the cell cycle [60]. The ER coregulator SRC3 functions as a coactivator of E2F1 thus has the potential to drive cell proliferation of BC cells [61]. CARM1 another ER coregulator regulates E2-stimulated BC growth through up-regulation of E2F, however, E2 stimulation of cyclin D1 is CARM1 independent [62]. The single molecule real-time (SMRT) coregulator functions as a dual coactivator and corepressor for ERα and participates in E2-induced progression through the G1/S transition of the cell cycle [63]. Mediator subunit 1 (MED1) [dementia-related psychosis (DRP) 205/tripartite ATP-independent periplasmic (TRAP) 220/percutaneous balloon pericardiotomy (PBP)] interacts with ER using its NR LxxLL motif and plays a role in the optimal expression of ER-dependent genes such as E2F1 and cyclin D1 which are known to promote progression through the G1/S phase of the cell cycle [64]. Ribosome biogenesis is linked to cell growth and proliferation with E2 signaling positively regulating rRNA synthesis [65]. Interestingly, PELP1 plays a critical role in ribosomal biogenesis and is needed for active ribosomal RNA transcription [66]. The PELP1 and its associated proteins, testis-expressed protein 10 (TEX10) and WD repeat domain 18 (WDR18), are involved in large ribosomal subunit maturation [67]. Furthermore, PELP1 is required for the optimum synthesis of the 60S ribosomal subunit [68]. Collectively, these findings suggest that coregulators play a critical role in driving E2-ERα cell cycle progression by promoting activation of cell cycle genes hence promoting ribosomal biogenesis (Figure 2).

## Coregulators in BC progression

Sustained exposure to E2 increases the risk of BC [28]. Deregulation of several coregulators involved in estrogen action has been reported in BC progression [69]. Specifically, this deregulation can drive the growth and progression of endocrine therapy resistant BC. Approximately 400 coregulators have been identified that can interact with ERα, and of those, approximately 100 coregulators are overexpressed in BC [70]. These alterations in the levels of coregulators enhance both ligand-independent, and ligand-dependent ERα signaling to drive growth and metastasis [69]. Recent studies utilizing next-generation sequencing of therapy-resistant and metastatic ERα+ BC have revealed that mutations in the *ESR1* gene (ERα) are frequent (30–40%) and contribute to acquired endocrine resistant BC [71–73]. Importantly, even these mtERs must interact with their coregulators to mediate ERα signaling [30]. Many ERα coregulator proteins are present at rate-limiting levels. Changes in the level of expression and/or activity of coregulators can provide growth advantages by enhancing ER signaling [74]. Some coregulators have the potential to function as master regulators and oncogenes [43]. For example, SRC3 is overexpressed and/or amplified in breast tumors [75]. Overexpression of SRC3/AIB1 promoted tumorigenesis in transgenic mouse models [76], while SRC3 knockout mouse models showed resistance in the initiation of tumorigenesis by both carcinogens and oncogenes [77]. A transgenic mouse model engineered with mammary gland PELP1 overexpression demonstrated that PELP1 deregulation will contribute to carcinoma of the mammary gland [78]. BC susceptibility gene 1 (BRCA1) functions as an ER corepressor and its mutations are correlated with an increased risk of BC [79]. The up-regulation of ERα corepressor MTA1, is associated with increased invasiveness and metastatic potential of BC [80]. In addition, oncogenic PELP1 signaling is implicated in the progression of BC [81]. Furthermore, PELP1 expression is upregulated during BC progression [82–84]. Overall, these findings suggest deregulation of ERα coregulators commonly occurs during BC progression; and changes in expression or function of these coregulators have the potential to contribute to endocrine therapy resistance.

## Coregulators and endocrine therapy resistance

Treatments for ERα+ BC involve blocking ER signaling with antiestrogens (AE) or aromatase inhibitors (AI). The selective ER modulator (SERM), tamoxifen was approved to treat BC by the Food and Drug Administration (FDA) in 1978. In 1996, the FDA approved drug anastrozole, an AI, was first utilized to treat hormone sensitive BC. In 2002, fulvestrant, an AI and selective ER degrader (SERD) were approved in the US as a therapeutic for ER+, HER2-advanced BC. Unfortunately, the majority of BC patients will eventually develop resistance to therapy, with progression to incurable metastases [85, 86]. While AE/AI are initially effective, *de novo* and/or acquired therapy resistance is common. Importantly, therapy resistant BC tumors retain their ER signaling which is mediated by the interactions between activated ERα and critical coregulator proteins [69, 87].

Alterations in the coregulator expression or functions enable ERα-signaling from AE-ER complexes, essentially converting the antagonist to an agonist [88, 89]. Approximately, 38% of *ESR1* coregulators identified in BC are over-expressed [87, 90, 91], such as SRC3 [92, 93], SRC2 [94], and PELP1 [95]. These deregulated coregulators contribute to BC progression [91], therapy resistance, and metastases [96–99]. The zeste homolog (EZH) 2-mediates epigenetic silencing of ERα cofactor growth regulation by estrogen in BC 1 (GREB1) contributes to the development of tamoxifen resistance [100]. As an adaptive response to endocrine therapy, tumors acquire mutations in the ERα-LBD [30, 101, 102]. These mtERα proteins have high constitutive transcriptional activity even in the absence of E2 [30, 71]. The constitutive activity of these mtERα proteins is strongly correlated with their ability to interact with coregulator proteins. Taken together, these data suggest that *ESR1* mutations in the LBD maintain the ERα-driven transcriptional program within these cancer cells, even in the absence of estrogenic ligand; thus contributing to endocrine resistance [30]. ERα mutations are also associated with estrogen insensitivity by affecting the coupling between ligand binding and coactivator recruitment [103]. A recent proteomics-based study suggested differential coactivator recruitment such as SRC1, 2, or 3 may be partly responsible for the ability of mtERα proteins to drive metastatic BC [104]. mtERα recruits coactivators in the absence of hormone; effectively conferring anti-estrogen resistance by modulating the dynamics of the loop connecting Helix 11 and Helix 12, thus giving rise to an altered antagonist state that resists inhibition [29].

Coregulators are implicated in the differential actions of SERMs [105]. Downregulation of expression of the ER corepressor, NCOR1, is associated with tamoxifen resistance [106]. The MTA1s variant inhibits ERα genomic activity by sequestering ERα in the cytoplasm [57]. Furthermore, altered localization of PELP1 in the cytoplasm results in activation of PI3K [107]. In addition, PELP1-containing complexes contribute to the outgrowth of stem-like cells associated with E2-independent BC progression [52]. Timeless is another ERα coactivator that promotes ERα-induced gene regulation through one of its proximal NR LxxLL motifs, enhances ERα poly-ADP-ribosylation (PARylation), and is implicated with tamoxifen resistance [108]. Collectively, these published studies indicate that ERα coregulator deregulation has the potential to promote ligand-independent ERα signaling. Therefore, targeting ERα coregulator functions could have potential therapeutic value in overcoming endocrine therapy resistance.

## Targeting coregulator functions in BC

**Figure 3.**
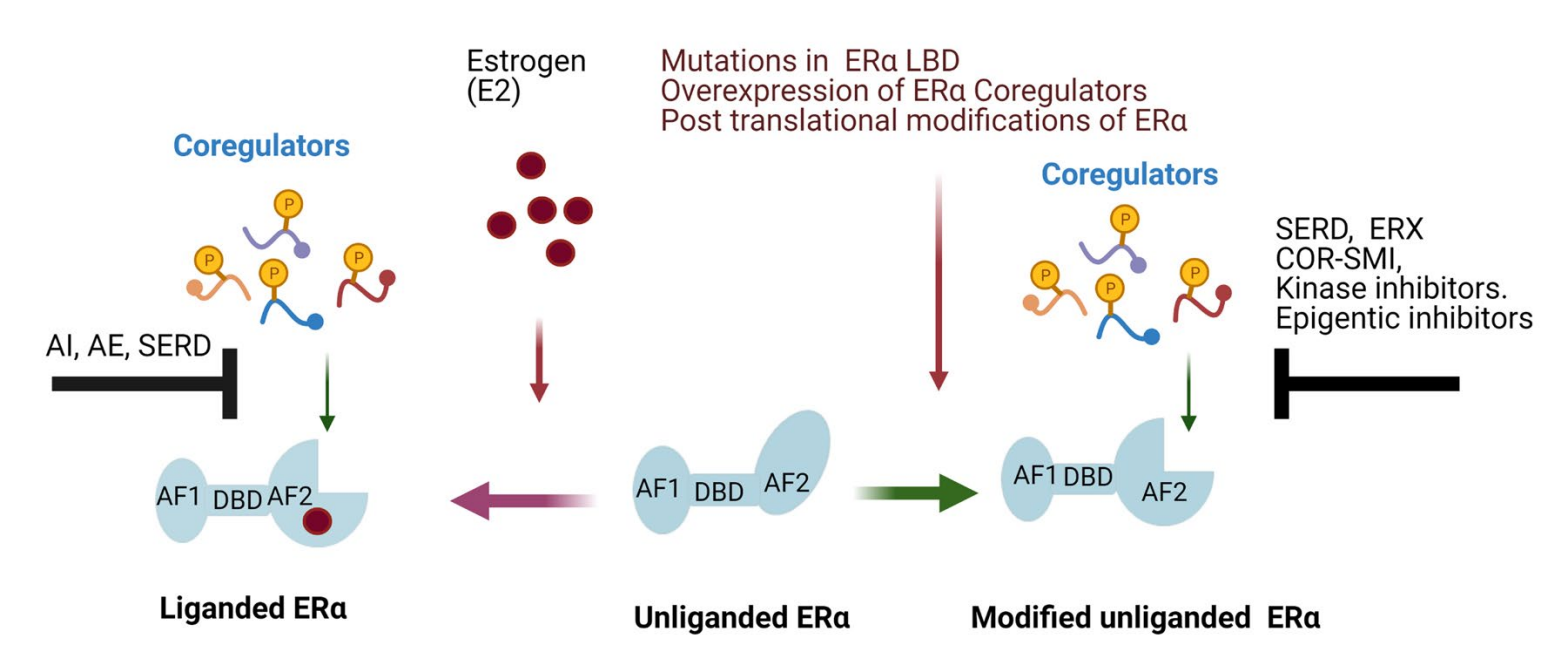
Approaches to target ERα-coregulator oncogenic signaling in BC. Ligand binding to the LBD of ERα facilitates coregulator recruitment and activation of ERα down-stream signaling. Ligand driven ERα-coregulator signaling can be targeted using AIs (such as letrozole), AEs (such as tamoxifen), and SERDs (such as fulvestrant). Cytosolic signaling kinases such as Src, AKT, mTOR, and CDKs form complexes with ERα; and can phosphorylate ER and its coregulators leading to activation of ERα non-genomic signaling cascades. Therefore, targeting these kinases with pharmacological inhibitors will be useful in reducing ERα coregulator driven non-genomic signaling. Several pathological scenarios such as overexpression of ERα coregulators, epigenetic changes, post translational modifications, and mutations in ERα AF2 domain can facilitate ligand-independent ERα signaling by recruiting coregulators. ERα signaling induced by oncogenic coregulators such as SRC1, SRC3, and PELP1 can be blocked by LxxLL motif blocking drugs called ERXs and SRC-3 small molecule inhibitor (SI-1) for SRC3 and inhibitors of epigenetic modifiers HDAC inhibitors (HDACi). Mutations in ERα create constitutive binding of coregulators to mtER and this signaling can be blocked by using ERXs or SMIs, targeting coregulators, or by utilizing SERDs to degrade mtERα. COR: coregulator

The contribution of multiple ER coregulators to endocrine therapy-resistant progression poses a therapeutic challenge, but also provides an opportunity for agents that specifically target oncogenic ER coregulators (Figure 3). Coregulators interact with ER via LxxLL motifs and blockage of these interactions may have therapeutic value. Early studies utilized LxxLL peptide-based approaches to target the ER-coregulator interface [109]. Progress in the area of LxxLL peptide-based inhibitors was recently reviewed by Skowron et al. [110]. A recent study used a cell-permeable stapled peptide, Arg4Lys1 (R4K1), to inhibit the ERα/coactivator axis interactions [111]. However, translating bioactive peptides as a potential therapeutic has pharmacological limitations. Recently, using a peptidomimetic strategy, a small chemical molecule that functions as an ER coregulator binding modulator (ERX)-11, was developed. ERX-11 blocks the interaction between a subset of coregulators with ERα. ERX-11 functions by blocking ER signaling and has also exhibited anti-proliferative activity against therapy-sensitive and therapy resistant human BC cells [112]. Furthermore, ERX-11 enhanced the efficacy of CDK4/6 inhibitor therapy and the combination of these two compounds may represent a viable therapeutic approach [113].

Steroid receptor coactivators (SRC1, 2, and 3) are implicated in ERα signaling and BC progression. Recent studies identified the cardiac glycoside, bufalin, as a potent SMI for SRC1 and SRC3. Bufalin promoted SRC3 protein degradation and reduced tumor growth in mouse xenograft models of BC [114]. Using a cell-based functional assay for high-throughput screening, Song et al. [115] identified the SMI, SRC-3 inhibitor-2 (SI-2), for SRC3. SI-2 selectively degraded SRC3 in cells through direct physical interactions with SRC3, and induced BC cell death with low nanomolar half-limiting dose (IC_50_) values, with no observed effects in the cell viability of normal cells. SI-2 represents a “first-in-class” drug that targets the oncogenic coactivator SRC3 and has great potential to treat advanced BC [115]. Screening for SRC SMIs also identified 4-Ethyl-2,6-bis-pyridin-3-ylmethylene-cyclohexanone (MCB-613), which functions as a potent SRC small molecule “stimulator”. This study found that MCB-613 selectively induced excessive stress in cancer cells implicating over-stimulation of the SRC coregulator signaling can be used as a potential strategy to kill cancer cells [116]. Unlike native [wild type (wt)] ERα, mtERα can bind to coregulators in the absence of ligand and contribute to endocrine therapy resistance by promoting ligand-independent ERα signaling. Therefore, SMIs targeting ERα coregulators could also be effective in targeting ligand-independent signaling by mtERα.

Another means to block aberrant coregulator signaling is to target mtERα using SERDs [117, 118]. Bazedoxifene (BZA), is a potent anti-estrogen that is shown to have improved inhibitory potency against mtERα compared to tamoxifen and also enhanced the efficacy of the CDK4/6 inhibitor, palbociclib [118]. Further, ER coregulators can contribute to ER crosstalk with endocrine signaling and metabolism which can alter downstream gene expression vital for tumor progression. Thus inhibitors targeting this axis will be useful in treating ER+ BC [119]. The mTOR axis is a critical component for PELP1 functions therefore, mTOR inhibitor(s) could be an agent for downregulating PELP1 oncogenic functions in BC [54]. Recent studies reported selective estrogen receptor covalent antagonist (H3B-5942), which can covalently inactivate both wt and mtERα by targeting cysteine (Cys) 530. H3B-5942 demonstrated significant activity as a monotherapy in xenograft models representing wtERα and mtERαY537S BC and was superior to the treatment with SERD, fulvestrant. Additionally, H3B-5942 potency was improved in combination with either CDK4/6 inhibitors and/or mTOR inhibitors in both wtERα and mtERα cell lines and/or tumor models [120].

Coregulator driven epigenetic changes are implicated in endocrine resistance. Since many coregulators have intrinsic and associated enzymatic activities, targeting these actions could represent another possible therapeutic strategy. HDACs inhibition has emerged as another potential strategy to overcome endocrine resistance especially in corepressor-deficient and tamoxifen-resistant BC [121]. The combination of the HDACi vorinostat and tamoxifen is well tolerated and exhibits encouraging activity in reversing hormone resistance. In addition, histone hyperacetylation is a useful pharmacodynamic marker for monitoring the efficacy of this combination therapy [122]. The ER coregulator PELP1 modulates epigenetic changes on ER target gene promoters via interactions with KDM 1, and KDM1 inhibitors are currently in clinical trials for other cancers [123]. KDM3A, a histone demethylase, is a positive regulator of ER activity and KDM3A deregulation contributes to endocrine therapy resistant disease [121]. EZH inhibitors have also been shown to overcome coregulator driven endocrine resistance in metastatic BC [100]. Considering the importance of coregulator signaling in BC progression and endocrine therapy resistance; SMIs that degrade coregulators, SMIs that interfere with coregulator binding to ER, or drugs that reverse coregulator driven epigenetic changes will have therapeutic value in treating endocrine therapy resistant BC.

## Conclusions

During the past two decades, significant progress was made in understanding the molecular basis of ERα signaling. These studies revealed a critical role of coregulators in both ERα genomic and non-genomic signaling. Further, published studies established that coregulator proteins play an integral role in endocrine therapy resistance in BC. A significant number of advanced ERα has driven breast tumors to contain either the modification of ERα by mutations that enhance coregulator binding or altered expression and functions of coregulator proteins. Future studies dedicated to elucidating the molecular mechanisms of coregulator signaling that occur in endocrine therapy resistant tumors are clearly needed. Development of oral SERDs that uniquely degrade mtERα or development of novel drugs that block mtERα interactions with coregulators will enable targeting of the ERα-coregulator signaling. An enhanced understanding of ER coregulator signaling that occurs in tumors will facilitate developing new combination therapy options for BC using small molecule drugs that target ERα-coregulator signaling.

## Abbreviations

AE: antiestrogens
AF: activation of function
AI: aromatase inhibitors
AIB1: amplified in breast cancer 1
BC: breast cancer
CARM1: coactivator-associated arginine methyltransferase 1
CDKs: cyclin-dependent kinases
E2: 17β-estradiol
E2F: early 2 factor
ER: estrogen receptor
ER+: ER-positive
EREs: estrogen response elements
ERX: estrogen receptor coregulator binding modulator
ERα: estrogen receptor alpha
ERα+: estrogen receptor alpha positive
ERβ: estrogen receptor beta
ESR: estrogen receptor
HDAC: histone deacetylase
HER2: human epidermal growth factor receptor-2
ILK: integrin-linked kinase
KDM: lysine-specific histone demethylase
LBD: ligand-binding domain
MAPK: mitogen-activated protein kinases
MTA1: metastasis-associated protein 1
mtER: mutant ER
mtERα: mutant ER alpha
mTOR: mechanistic target of rapamycin
NR: nuclear receptor
PELP1: proline glutamic acid and leucine-rich protein 1
PI3K: phosphatidylinositol 3-kinase
PR: progesterone receptor
SERD: selective estrogen receptor degrader
SMIs: small molecule inhibitors
SRC: steroid receptor coactivator
TNBC: triple-negative breast cancer
TRE: trehalose
wt: wild type

## Declarations

### Copyright

© The Author(s) 2021.
